# Application of geometric methods to the morphological classification of five abalone species

**DOI:** 10.3897/BDJ.14.e201558

**Published:** 2026-08-12

**Authors:** Ying Tian, Wenjia Li, Jiancheng Zhang, Wanle Sun, Qingqiao Li, Sining Hou, Zhenlin Hao

**Affiliations:** 1 Key Laboratory of Mariculture & Stock Enhancement in North China Sea, Ministry of Agriculture and Rural Affairs, Dalian Ocean University, Dalian, China Key Laboratory of Mariculture & Stock Enhancement in North China Sea, Ministry of Agriculture and Rural Affairs, Dalian Ocean University Dalian China; 2 Beisen Seafood (DaLian)Co.,Ltd, Dalian, China Beisen Seafood (DaLian)Co.,Ltd Dalian China

**Keywords:** aquaculture, geometric morphometrics, Haliotidae, morphological classification

## Abstract

Abalone is an economically important mollusc in the aquaculture industry; however, research on the morphological classification and geographical origin tracing of abalone species remains limited. In the present study, a total of 177 specimens were collected, comprising five abalone species — *Haliotis madaka* (T. Habe, 1977), *Haliotis diversicolor* Reeve, 1846, *Haliotis gigantea* Gmelin, 1791, *Haliotis ovina* Gmelin, 1791 and *Haliotis discus hannai* Ino, 1953 (including samples from wild and cultured populations in Liaoning Province, as well as a cultured population in Fujian Province). Generalised Procrustes Analysis (GPA) was applied to standardise the landmark data, thereby removing non-morphological sources of variation. Subsequently, multivariate statistical methods — including Principal Component Analysis (PCA) and Canonical Variate Analysis (CVA) were employed to quantify morphological differences.

PCA results showed that PC1 and PC2 accounted for 62.23% and 12.68% of the total morphological variance amongst the five species, respectively, with a cumulative contribution of 74.91%. For the three *H. discus hannai* populations (Liaoning-wild, Liaoning-cultured and Fujian-cultured), PC1 and PC2 explained 50.57% and 12.07% of the total variance, respectively. CVA revealed that the first two canonical variates accounted for 69.59% of the overall shape variation amongst groups. These findings demonstrate that geometric morphometric approaches effectively distinguish amongst the five abalone species examined and successfully differentiate between wild and cultured specimens of *H. discus hannai*. However, the two cultured populations from Liaoning and Fujian Provinces exhibited considerable morphological overlap along the primary discriminant axis, precluding complete separation.

## Introduction

Abalone belongs to the phylum Mollusca, class Gastropoda, order Archaeogastropoda, family Haliotidae and genus *Haliotis*. The family Haliotidae comprises a single genus, with approximately 76 extant species currently recognised and 25 species documented in the fossil record (MolluscaBase 2026). Abalone represents one of the most economically important molluscs in the global aquaculture industry. In recent years, global aquaculture production of abalone has increased substantially, while wild-caught harvests have declined. Global wild-caught abalone yields approached 20,000 tonnes during the 1970s, but decreased to approximately 5,850 tonnes in the 2020/21 fishing season (Cook 2023). Amongst the cultivated abalone species worldwide, the Pacific abalone (*Haliotis discus hannai* Ino, 1953) dominates global production, particularly in China and the Republic of Korea. Japan also cultivates *H. discus hannai*, *Haliotis madaka* (T. Habe, 1977) and *Haliotis gigantea* Gmelin, 1791 (Geiger and Groves 2015).

As the primary producer and consumer of abalone globally, China's aquaculture production reached 203,500 tonnes in 2020, accounting for nearly 90% of total worldwide output (Lin et al. 2020). According to the 2025 China Fishery Statistical Yearbook, abalone ranks as the second most important marine-farmed mollusc species in China, with the cultivated area expanding to 17,582 hectares and production reaching 252,000 tonnes — a 23.83% increase compared to 2020 (Bureau of Fisheries, Ministry of Agriculture and Rural Affairs of the People's Republic of China 2025). The Pacific abalone (*H. discus hannai*) is the dominant species in Chinese aquaculture, primarily distributed along the coastal regions of the Liaodong Peninsula, Shandong Peninsula (Yu et al. 2012). Major production regions within China include Liaoning and Fujian Provinces (Zhang 2019), with notable quality variations observed amongst specimens from different localities. For instance, abalone from southern regions exhibit higher average hardness and chewiness in the foot muscle, whilst northern specimens demonstrate superior elasticity (Yu et al. 2012).

The continued expansion of China's abalone aquaculture industry has brought forth considerable challenges. Large-scale production has been accompanied by issues including germplasm degradation resulting from prolonged inbreeding, miniaturisation of individuals, reduced stress resistance and population mixing due to inadequate management practices. These factors have substantially compromised the ability to determine population origin. Consequently, the development of non-destructive methods for identifying parental stocks and rapidly selecting abalone families for selective breeding programmes (Luan et al. 2022) has emerged as an urgent priority for improving abalone production performance.

Geometric morphometric methods have been widely applied in molluscan research. Early studies predominantly employed two-dimensional (2D) imaging combined with geometric morphometric approaches. Márquez et al. (2010) effectively distinguished amongst different phenotypic stages of the Patagonian scallop (*Aequipecten tehuelchus*) across various growth phases using geometric morphometric techniques. Cruz et al. (2012) applied geometric morphometric analysis to selected species within the family Conidae using 22 coordinate landmarks, proposing that the aperture and spire regions constitute significant diagnostic features for species identification within this family. Lai et al. (2016) analysed two colour morphs of the giant apple snail (*Pomacea maculata*) using geometric morphometric methods, revealing that morphological variation was primarily concentrated in the upper portion of the aperture. Abdelhady et al. (2018) conducted a geometric morphometric investigation of *Melanoides tuberculata* from Lake Manzala, Egypt, demonstrating that specimens from polluted environments exhibited reduced morphological variation, with heavy metals including lead, zinc, chromium and copper identified as primary predictors of shell morphology. Chen et al. (2018) examined morphological differences and phylogenetic relationships amongst *Littorina scabra*, *L. brevicula*, *Nodilittorina exigua* and *N. pyramidalis* using landmark and semi-landmark approaches. Miranda (2020) performed geometric morphometric analysis on the land snail *Bostryx torallyi* collected from Bolivia and Argentina, concluding that geometric morphometric methods represent an effective approach for evaluating shell shape variation across geographical regions. Shu et al. (2021) conducted morphometric studies and comparative analyses of the zhikong scallop (*Chlamys farreri*) from varying water depths, indicating that farmed specimens from shallow waters exhibited smaller shell dimensions than those from deeper waters.

Current approaches to abalone classification primarily encompass molecular genetic methods, chemical marker analysis and traditional morphological identification (Geiger and Groves 2015). Brown (1993) successfully distinguished 17 abalone species using protein gel electrophoresis. Lee and Vacquier (1995) employed abalone sperm lysin sequences for taxonomic classification. Coleman and Vacquier (2014) applied internal transcribed spacer (ITS) sequence analysis. Wang et al. (2017) utilised cytochrome oxidase I (COI) gene sequences for species identification. Hong et al. (2023) differentiated abalone populations, based on strontium (Sr) stable isotope ratios and mineral element compositions. Nevertheless, DNA-based identification methods present considerable limitations, including high operational costs and the requirement for destruction of soft tissue, rendering them unsuitable for family selection during aquaculture operations.

Zhang et al. (2022) demonstrated that variations in geographical origin and growth rate induce changes in shell morphology of *H. discus hannai*, with shell morphological characteristics exhibiting strong linear correlations with allometric growth differences amongst populations. Accordingly, the diverse environmental and ecological conditions along China's coastal regions may substantially influence phenotypic plasticity, whilst artificial seeding practices could introduce additional genetic effects. Estes et al. (2005) further noted that the evolution of abalone body shape appears correlated with primary productivity and foraging strategies. Variations in seawater temperature and salinity may affect growth and metabolic processes in *H. discus hannai*, consequently influencing growth rates, survival rates and, ultimately, shell morphology (Wei et al. 2018).

Traditional morphological classification typically involves initial size-based selection according to shell length, followed by differentiation, based on shell shape characteristics, surface sculpture and number of respiratory pores (Zhang et al. 2022). However, the considerable morphological variability exhibited by abalone shells renders traditional classification approaches prone to substantial errors, requiring extensive manual comparison. Furthermore, such methods fail to effectively distinguish between specimens of the same species originating from different habitats. Zhang (2019) applied geometric morphometric analysis to *Haliotis fulgens* Philippi, 1845, *Haliotis rufescens* Swainson, 1822, *H. discus hannai* Ino, 1953, varied abalone (*Haliotis diversicolor* Reeve, 1846) and *Haliotis sieboldii* Reeve, 1846, successfully achieving complete separation amongst all five species and providing a novel quantitative framework for abalone morphological research (Zhang 2019).

The present study aims to apply geometric morphometric analysis to distinguish economically important abalone species and different populations of *H. discus hannai*, based on shell morphology and to effectively differentiate *H. discus hannai* specimens according to geographical origin and cultivation method.

## Material and methods

### Sample collection

**Figure 1.**
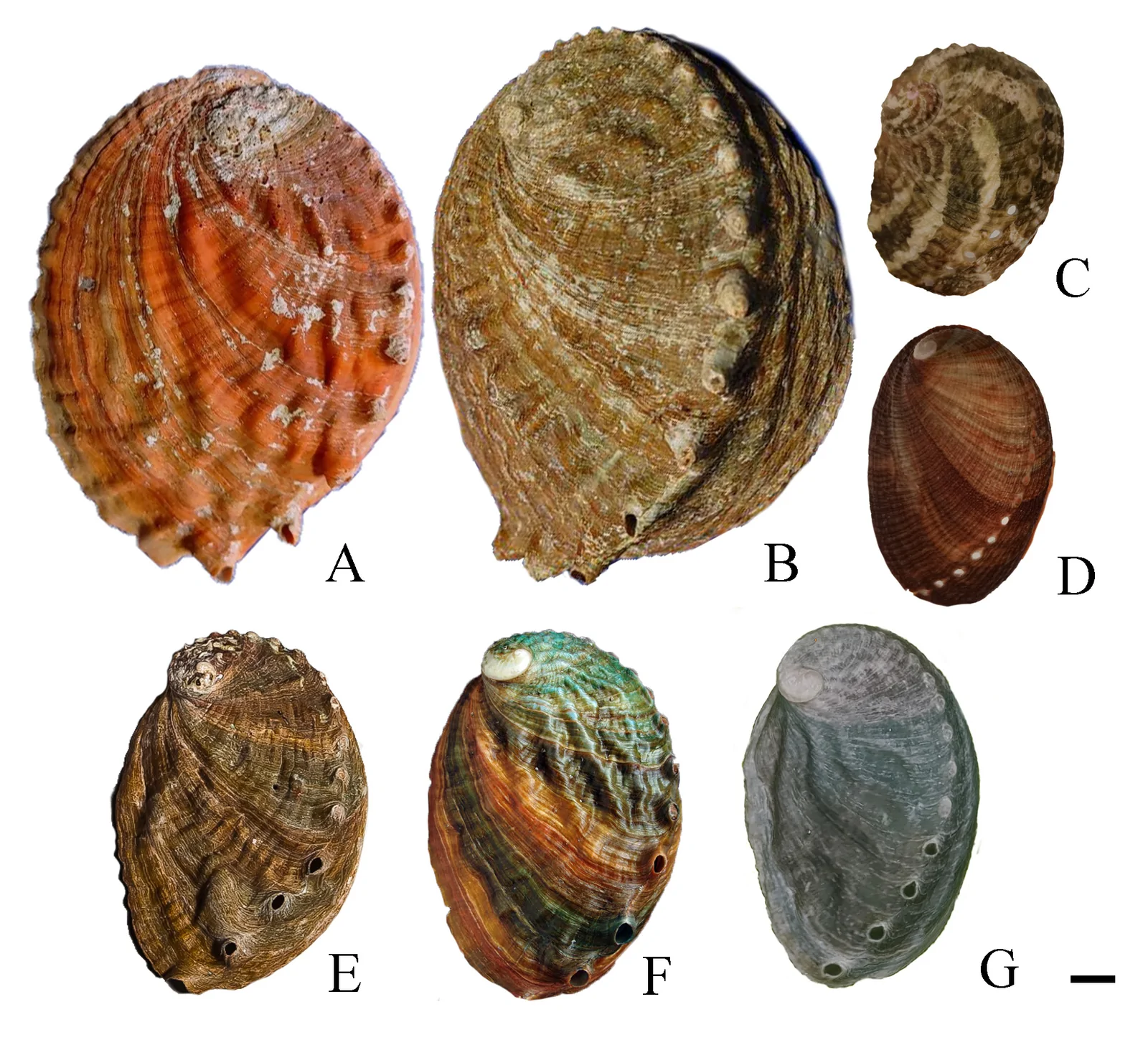
Five abalone species examined in this study. **A** *Haliotis gigantea* Gmelin, 1791; **B** *Haliotis madaka* (T. Habe, 1977); **C** *Haliotis ovina* Gmelin, 1791; **D** *Haliotis diversicolor* Reeve, 1846; **E** *Haliotis discus hannai* Ino, 1953 (Liaoning wild); **F** *Haliotis discus hannai* Ino, 1953 (Fujian cultured); **G** *Haliotis discus hannai* Ino, 1953 (Liaoning cultured）(scale bars = 1 cm).

A total of 177 abalone specimens were collected for this study, comprising five species — *Haliotis madaka* (T. Habe, 1977), *Haliotis diversicolor* Reeve, 1846, *Haliotis gigantea* Gmelin, 1791, *Haliotis ovina* Gmelin, 1791 and *Haliotis discus hannai* Ino, 1953 — including three populations of *H. discus hannai* from two geographical origins (Table 1 and Fig. 1). Wild specimens of *H. discus hannai* were collected from the coastal waters off Dalian, Liaoning Province, China. Cultured specimens were obtained from raft-cultured populations in Dalian, Liaoning Province and raft-cultured populations in Lianjiang County, Fuzhou, Fujian Province. All specimens were collected between 2023 and 2024. Following collection, specimens with incomplete shells or damaged margins were excluded to ensure a final sample set comprising only specimens with intact marginal regions. Specimens were classified and identified according to geographical origin and shell morphology, following the criteria described by Geiger and Groves (2015). Shell length measurements of the five species are summarised as follows: *Haliotis madaka* (137 ± 8.2 mm); *H. gigantea* (132.8 ± 8.7 mm); *H. diversicolor* (56.38 ± 7.9 mm); *H. ovina* (45.29 ± 5.3 mm); and *H. discus hannai* (44.62 ± 9.6 mm). For the three *H. discus hannai* populations, shell length measurements were also recorded: wild specimens from Liaoning Province (43.1 ± 6.3 mm); cultured specimens from Liaoning Province (46.7 ± 4.9 mm); and cultured specimens from Fujian Province (62 ± 9.6 mm).

### Image Acquisition

**Figure 2.**
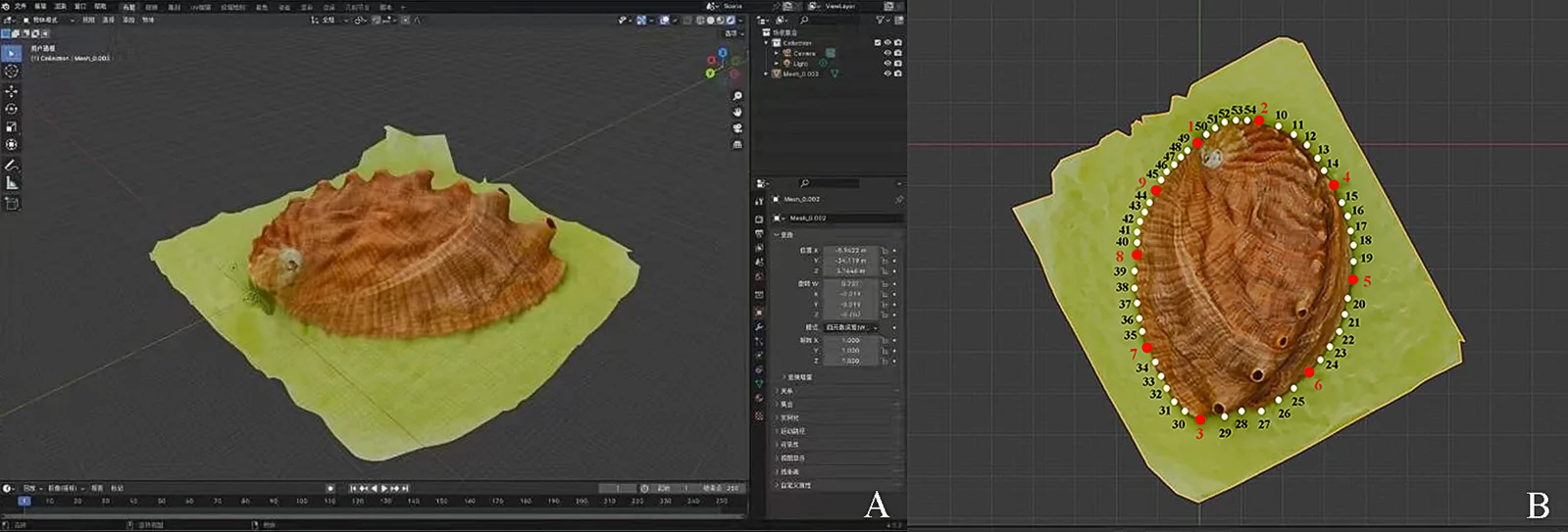
3D image acquisition and landmark demonstration of *Haliotis*.

Digital images of abalone shell were captured using a Canon G12 digital camera, positioned with the shell surface facing upwards. A reproduction stand was employed to maintain a constant distance between the camera lens and each specimen. Images were captured from multiple angles — anterior, posterior, lateral and dorsal views — with a minimum of 80 photographs obtained per specimen. Three-dimensional mathematical models were reconstructed from the captured images using 3DF Zephyr software. Minor refinements to the models were performed using Blender software and standardised shell shape data from consistent viewing angles were extracted by identifying corresponding pixel coordinates across images. Landmark coordinates were subsequently digitised using ImageJ to obtain the raw morphometric data (Fig. 2A).

Although the final morphometric analysis is based on two-dimensional (2D) landmark coordinates, a 3D modelling workflow was adopted for two principal reasons. First, abalone possess a single, asymmetrically coiled shell that cannot be captured in its entirety from any single photographic angle; the 3D reconstruction enables comprehensive visualisation and standardisation of shell morphology across all specimens. Second, the 3D models allow re-orientation of the shell to a consistent anatomical reference frame prior to landmark digitisation. This ensures that the 2D landmark coordinates extracted from standardised viewing planes are directly comparable amongst specimens, thereby eliminating positional variation attributable to differences in specimen placement during photography. By contrast, direct 2D photography of shells in fixed orientations would be susceptible to inconsistencies in mounting angle and perspective, potentially introducing systematic bias into the landmark data and compromising the reliability of subsequent morphometric analyses.

### Standardisation of data

Using landmarks and semi-landmarks, geometric morphometric standardisation was performed on the captured abalone shell images. A total of 54 points, including nine landmarks and 45 semi-landmarks, were selected as markers for geometric morphometric standardisation (Fig. 2B). The selection of landmarks and semi-landmarks followed the criteria described by Bookstein (1997), Márquez et al. (2010) and Mitteroecker and Gunz (2013), focusing on the external contour, apex and marginal features of the abalone shell.

The landmarks are defined as follows:

(1) shell apex; (2) upper inflection point of the posterior margin; (3) most convex point of the ventral margin; (4) mid-point of the right anterior margin; (5) mid-point of the right ventral margin; (6) characteristic point near the respiratory pores; (7) mid-point of the left ventral margin; (8) mid-point of the left anterior margin; (9) upper point of the left posterior margin.

Semi-landmarks were placed along the shell contour as follows:

(10–18) along the right anterior margin contour; (19–27) along the right ventral to posterior margin contour; (28–36) along the posterior to ventral margin contour; (37–45) along the left ventral to anterior margin contour; (46–54) along the left anterior margin to shell apex contour.

### Data processing and Analysis

GPA was applied to landmark coordinates to remove non-morphological variation attributable to scale, orientation and position. GPA transforms and rotates landmark data to a common origin and scales them to unit centroid size. The GPA-transformed coordinates were subsequently employed as morphological variables for further analysis. Principal Component Analysis (PCA) and Canonical Variate Analysis (CVA) were performed to assess shell shape differences amongst the abalone species. All data processing and statistical analyses were conducted using ImageJ, PAST 4.17 and R 4.4.3.

## Results

### Principal component analysis

**Figure 3.**
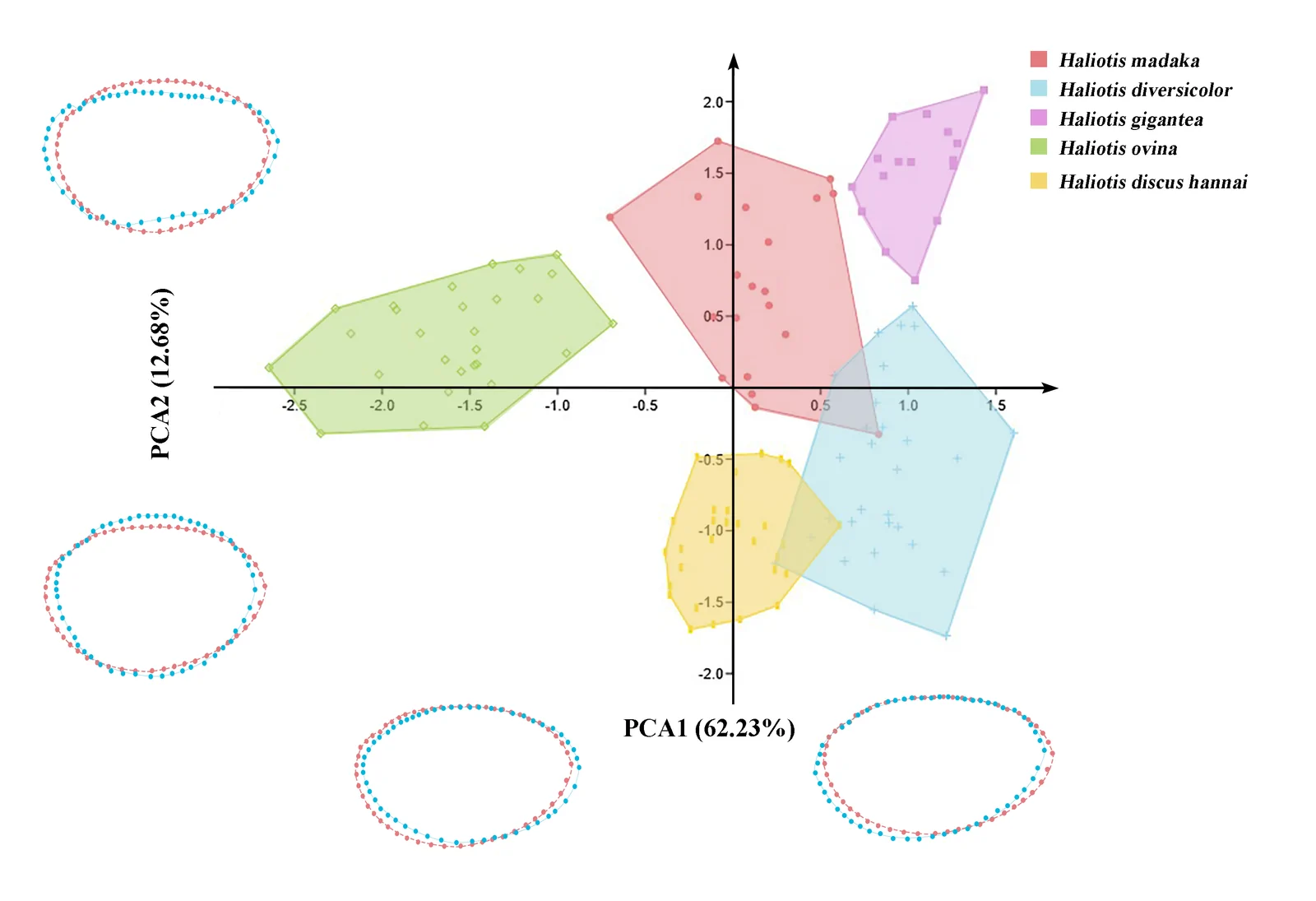
Principal Components Analysis (PCA) diagram of five abalones.

Principal Component Analysis (PCA) was applied to the geometric morphometric data of the five abalone species for dimensionality reduction and visualisation. The results showed that the first principal component (PC1) and second principal component (PC2) explained 62.23% and 12.68% of the morphological variance, respectively, with a cumulative contribution of 74.91%. These findings indicate that these two components effectively capture the primary morphological variation in abalone shell shape (Fig. 3).

Regarding the direction of variation along PC1, this component primarily reflects changes in the width-to-length ratio of the shell. Positive scores correspond to elongated, narrow shell discs with relatively smaller landmarks and semi-landmarks (LM2, LM3 and LM14–18), whilst negative scores are associated with flattened shell morphology. PC2 primarily characterises variations in shell disc ellipticity, with positive scores indicating elliptical shell shapes and negative scores representing more circular morphology.

The PCA scatterplot demonstrates distinct interspecific differentiation amongst the five abalone species examined. *H. ovina* occupies the negative region of PC1, forming a pronounced spatial separation from the other four species, thereby indicating substantial morphological divergence of this species in shell shape. *H. madaka* and *H. gigantea* are distributed in the positive regions of both PC1 and PC2, with *H. gigantea* exhibiting a more concentrated distribution in the upper-right quadrant. Both *H. discus hannai* and *H. diversicolor* are positioned in the positive region of PC1 and the negative region of PC2, with considerable spatial overlap, suggesting high morphological similarity between these two species, based on the geometric morphometric variables measured.

**Figure 4.**
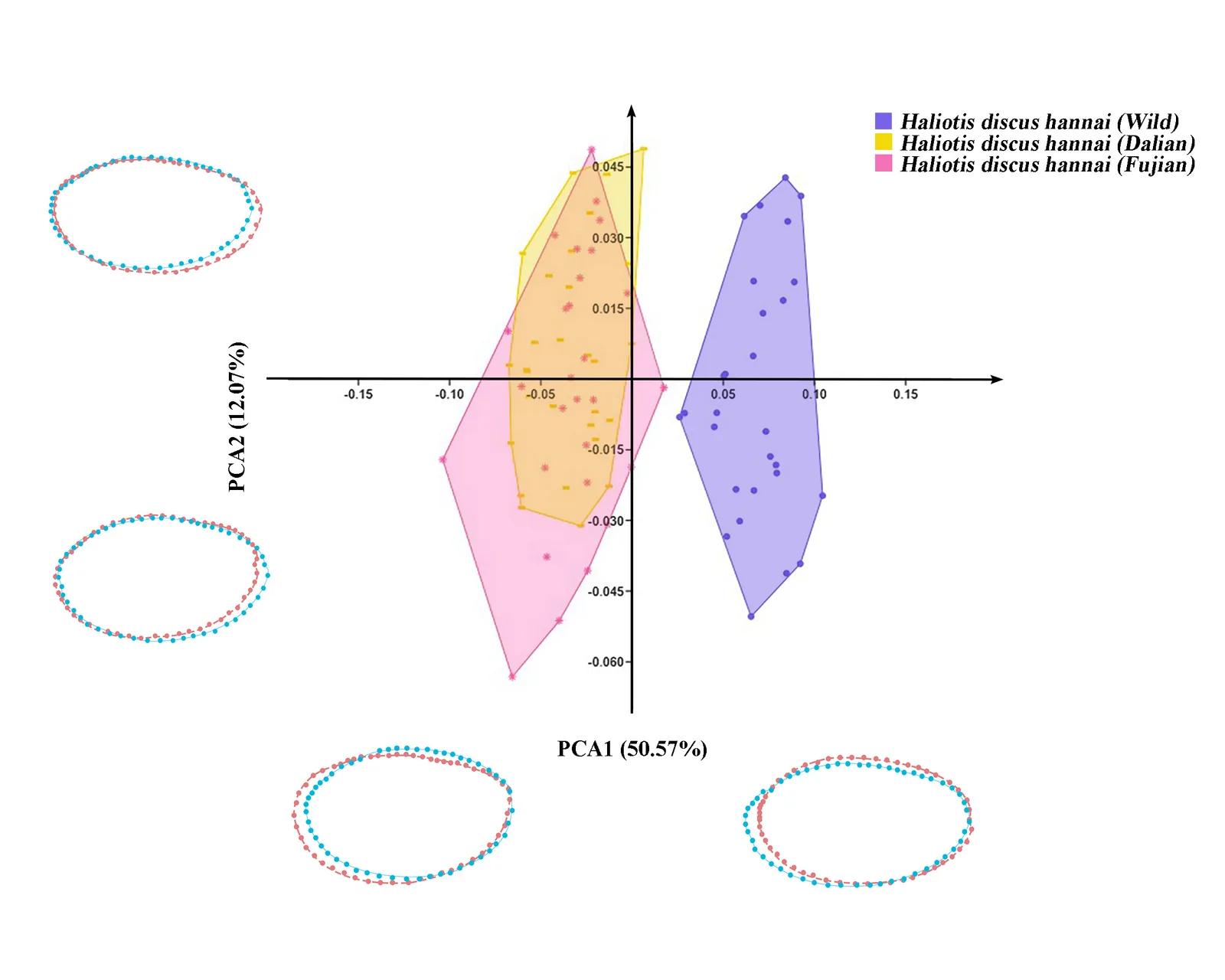
Principal Component Analysis (PCA) ordination of *H. discus hannai* from three populations: wild specimens from Liaoning (purple), cultured specimens from Dalian, Liaoning (yellow) and cultured specimens from Fujian (pink).

PCA was applied to the morphological measurement data from three *H. discus hannai* populations (wild, Dalian-cultured and Fujian-cultured), as illustrated in Fig. 4. The PC1 and PC2 explained 50.57% and 12.07% of the total variance, respectively, representing a cumulative total of 62.64% of the observed morphological variation.

Within the PCA ordination space, the three populations displayed distinct clustering patterns. The wild population (depicted in purple) was primarily distributed in the positive region of PC1 (0.03 — 0.13), demonstrating complete separation from the two cultured populations along this axis. The Fujian-cultured population (depicted in pink) formed the largest and most dispersed cluster, occupying the negative region of PC1 (–0.13 — –0.02) with considerable vertical dispersion along PC2 (–0.06 — –0.05). The Dalian-cultured population (depicted in yellow) was positioned in the negative-to-zero region of PC1 (–0.08 — –0.01), biased towards positive values along PC2 (–0.03 — –0.05).

The PCA results indicate that PC1, functioning as the primary discriminant axis, effectively distinguishes wild specimens from cultured ones, reflecting the substantial influence of habitat environment (natural marine versus aquaculture conditions) on the morphological characteristics of *H. discus hannai*. The considerable overlap between the two cultured populations along PC1 suggests high morphological similarity in their principal shell shape features. PC2 reveals within-population variation patterns, with the Fujian-cultured population exhibiting greater morphological diversity. Furthermore, the morphological variation diagrams positioned around the periphery of the figure provide an intuitive representation of the gradual shell shape transformation along the PC1 axis.

### Canonical Variate Analysis

**Figure 5.**
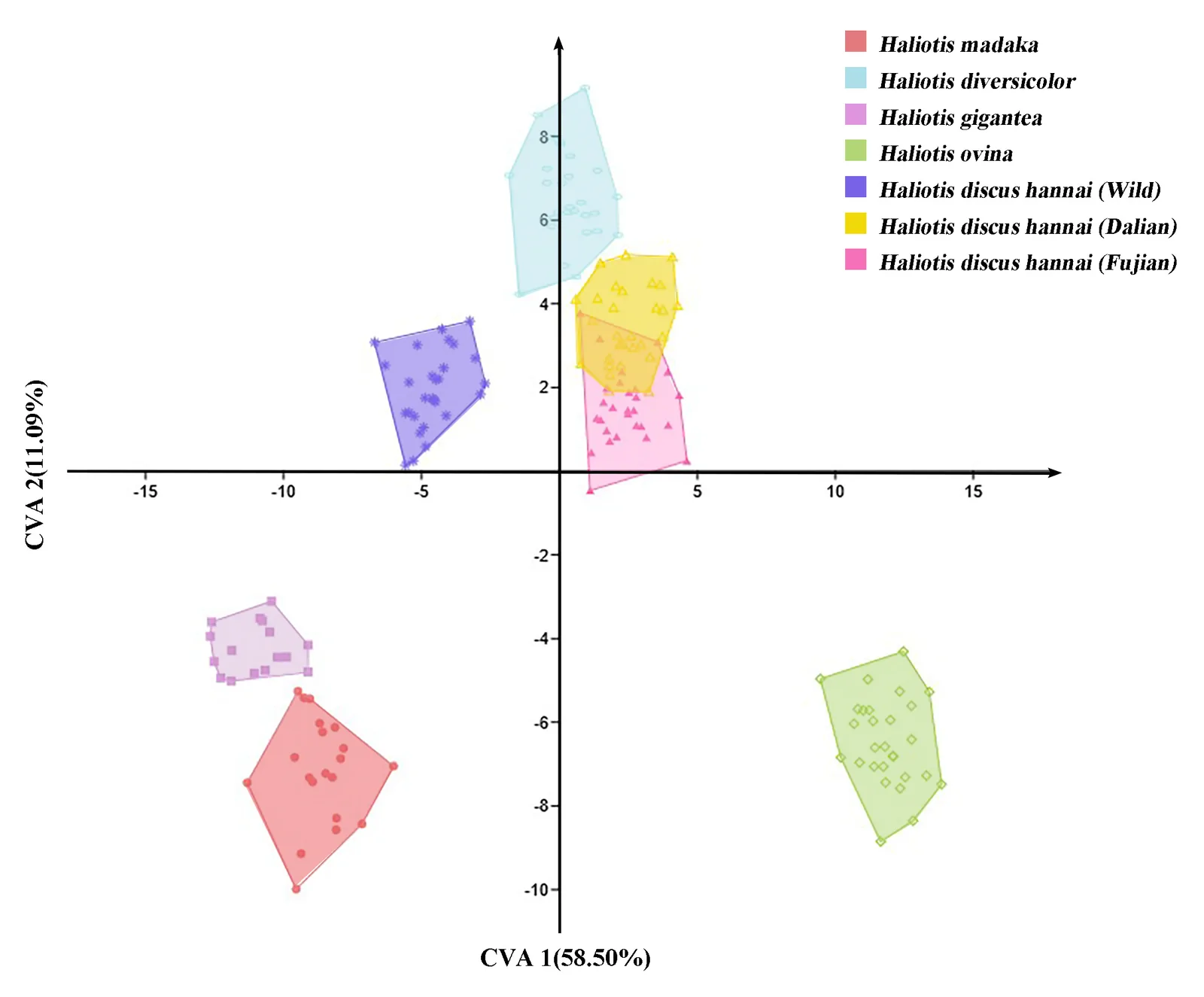
Canonical Variate Analysis (CVA) of five abalone species and three distinct populations of *Haliotis discus hannai*.

Canonical Variate Analysis (CVA) was applied to the five abalone species to evaluate the reliability of geometric morphometric methods for species identification. The first linear discriminant function (CV1) and second linear discriminant function (CV2) explained 58.5% and 11.09% of the total between-group variance, respectively, representing a cumulative contribution of 69.59%. These findings indicate that the discriminant model effectively captures the amongst-group variation inherent in the original morphological data (Fig. 5).

The CVA ordination plot demonstrates complete separation amongst the five abalone species within the two-dimensional discriminant space, with no substantial overlap between groups. This confirms the efficacy and accuracy of geometric morphometric methods for abalone species identification. *H. discus hannai* is tightly clustered near the origin of the coordinate system, indicating that its morphological characteristics are representative of the overall sample mean. This species exhibits the smallest morphological variation amongst the five species examined. *H. gigantea* occupies the negative region of CV1 and positive region of CV2, whilst *H. madaka* is positioned in the negative region of both CV1 and CV2. Although these two species cluster together along CV1, they demonstrate significant separation along CV2, suggesting overall similarity in general shell contour morphology, but distinguishable differences in secondary characteristics such as shell disc ellipticity. *H. diversicolor* occupies the positive region of both CV1 and CV2, whereas *H. ovina* is positioned in the positive region of CV1 and negative region of CV2. The contrasting distribution of these species along CV1 indicates that their morphological differentiation is primarily attributable to variation in shell width-to-length ratio.

Regarding the different *H. discus hannai* populations examined, the wild population exhibits a complete negative shift along CV1 (-7 — -3), forming a distinct separation boundary from the two cultured populations within the PCA ordination space. The two cultured populations demonstrate substantial overlap along CV1 (0 — 5), suggesting that, although these populations originate from geographically distant regions, shared cultivation practices may impose similar morphological selection pressures. Nevertheless, differences are observed along CV2 between these populations: the Dalian population is biased towards positive values along CV2 (2 — 5), whilst the Fujian population exhibits a broader range of variation along CV2 (1— 4).

### Jackknife analysis

**Figure 6.**
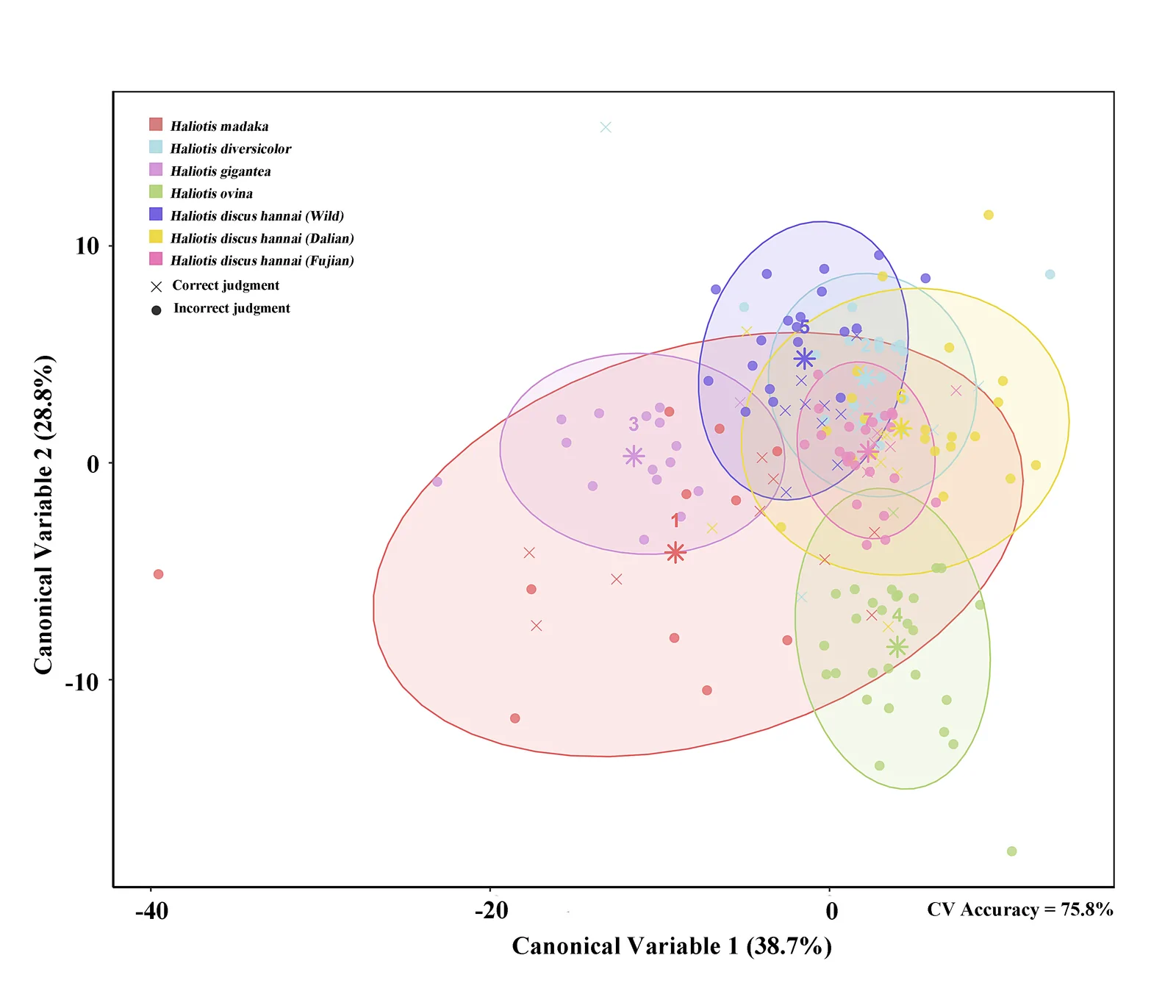
Jackknife Analysis of five abalone species and three distinct populations of *Haliotis discus hannai*.

Linear discriminant functions were constructed using the first two principal components derived from the CVA and validated through a Jackknife analysis. The results indicate that the discriminant functions correctly classified 74.91% of abalone specimens to their respective species and 62.64% to their geographical populations. The Jackknife analysis classification accuracy reached 75.8%, thereby confirming the efficacy of the discriminant functions (Fig. 6).

## Discussion

Previous research has predominantly focused on traditional morphometric parameters, such as shell length, shell width and shell height, while comparative studies examining inter-specific differences amongst economically important abalone species remain relatively limited. The complete separation of the five abalone species in the PCA space can be fundamentally attributed to their distinct geographic ranges and ecological niches and this ecological reality has directly shaped their unique shell morphology.

Amongst these species, *H. ovina* occupies an extreme position in the negative PC1 region, forming a pronounced spatial distinction from all other species. Notably, *H. ovina* exhibits an elliptical body shape and represents the sole tropical species amongst the five taxa, demonstrating a marked morphological divergence from the elongated forms characteristic of the remaining four species. This differentiation reflects the unique ecological strategies that have developed during the long-term evolutionary process. Although these five abalone species belong to the same genus, prolonged geographic isolation has led to independent morphological evolution.The principal advantage of PCA as a dimensionality reduction technique lies in its capacity to transform these subtle morphological differences into visualised spatial separation patterns. The discriminant function analysis achieved a correct classification rate of 74.91%, demonstrating that geometric morphometrics can serve as an effective method for species identification.

Environmental factors, including temperature, salinity and nutritional status, exert significant effects on the shell morphology (Telesca et al. 2019). Geographic isolation has been reported to strongly influence shell morphology (Krapivka et al. 2007). However, in the present study, substantial overlap was observed between the *H. discus hannai* populations from Fujian and Liaoning Provinces, indicating morphological similarity that renders geometric morphometric separation challenging.

The Pacific abalone (*H. discus hannai*) is primarily distributed along the coastal regions of the Liaodong Peninsula, Shandong Peninsula and northern Jiangsu Province in China (Yu et al. 2012). The optimal growth temperature for adult abalone ranges from 10 to 22°C, with mortality frequently occurring when water temperature exceeds 26°C or falls below 0°C (Zhang et al. 2005). Given the cold winters in northern China, indoor overwintering requires heating of seawater, which consumes substantial energy resources (Nie et al. 1996). To reduce costs, some hatchery operators have begun experimenting with overwintering in southern sea areas and this approach has received industry endorsement (Wu et al. 2009).

As a species exhibiting pronounced phenotypic plasticity, the morphological characteristics of *H. discus hannai* are largely regulated by environmental conditions. Although environmental factors, such as seawater temperature and salinity, differ between Dalian and Fujian, both populations are maintained under human-managed aquaculture conditions, which inherently produce a homogenising effect. The high-density, intensive management practices characteristic of aquaculture environments effectively neutralise the geographic isolation effects observed in natural settings. Regardless of geographic location, abalone in both Dalian and Fujian undergo similar aquaculture cycles, feeding regimes and spatial density conditions. Under these uniform artificial selection pressures, both populations have evolved towards similar morphological trajectories, resulting in convergent morphological characteristics across geographically distinct populations.

## Figures and Tables

**Table 1.** The information on abalone samples.

**Species**	**Locality**	**number**
*Haliotis madaka* (T. Habe, 1977)	Honshu, Japan	22
*Haliotis diversicolor* Reeve, 1846	Guangxi, China	22
*Haliotis gigantea* Gmelin, 1791	Tohoku, Japan	21
*Haliotis ovina* Gmelin, 1791	Xisha Islands, China	22
*Haliotis discus hannai* Ino, 1953	Liaoning, China (Raft-cultured)	30
*Haliotis discus hannai* Ino, 1953	Fujian, China (Raft-cultured)	30
*Haliotis discus hannai* Ino, 1953	Liaoning, China (wild)	30
